# PET Imaging of GPR44 by Antagonist [^11^C]MK-7246 in Pigs

**DOI:** 10.3390/biomedicines9040434

**Published:** 2021-04-16

**Authors:** Pierre Cheung, Bo Zhang, Emmi Puuvuori, Sergio Estrada, Mohammad A. Amin, Sofie Ye, Olle Korsgren, Luke R. Odell, Jonas Eriksson, Olof Eriksson

**Affiliations:** 1 Science for Life Laboratory, Uppsala University, 751 83 Uppsala, Sweden; pierre.cheung@ilk.uu.se (P.C.); bo.zhang@ilk.uu.se (B.Z.); emmi.puuvuori@ilk.uu.se (E.P.); 2 Department of Medicinal Chemistry, Uppsala University, 751 83 Uppsala, Sweden; sergio.estrada@ilk.uu.se (S.E.); mohammad.amin@ilk.uu.se (M.A.A.); sofie.ye@ilk.uu.se (S.Y.); luke.odell@ilk.uu.se (L.R.O.); 3 Department of Immunology, Genetics and Pathology, Uppsala University, 751 83 Uppsala, Sweden; olle.korsgren@igp.uu.se

**Keywords:** pancreas imaging, diabetes, biomarker, PET, PTGDR2

## Abstract

A validated imaging marker for beta-cell mass would improve understanding of diabetes etiology and enable new strategies in therapy development. We previously identified the membrane-spanning protein GPR44 as highly expressed and specific to the beta cells of the pancreas. The selective GPR44 antagonist MK-7246 was radiolabeled with carbon-11 and the resulting positron-emission tomography (PET) tracer [^11^C]MK-7246 was evaluated in a pig model and in vitro cell lines. The [^11^C]MK-7246 compound demonstrated mainly hepatobiliary excretion with a clearly defined pancreas, no spillover from adjacent tissues, and pancreatic binding similar in magnitude to the previously evaluated GPR44 radioligand [^11^C]AZ12204657. The binding could be blocked by preadministration of nonradioactive MK-7246, indicating a receptor-binding mechanism. [^11^C]MK-7246 showed strong potential as a PET ligand candidate for visualization of beta-cell mass (BCM) and clinical translation of this methodology is ongoing.

## 1. Introduction

A key hallmark of both type 1 and type 2 diabetes is loss of beta-cell mass (BCM) and function (BCF). With current methodologies, BCM can only be studied by staining of postmortem biopsies, which precludes longitudinal assessment [1]. Current plasma markers such as blood glucose concentration, percentage of glycated hemoglobin (HbA1c), and C-peptide levels only give an estimation of BCF instead of BCM, again making it challenging to longitudinally estimate the residual BCM in patients with type 1 or type 2 diabetes—both during the progression of disease and in response to therapy.

Positron-emission tomography (PET) is a highly sensitive and quantitative medical imaging technique that was proposed to noninvasively image the pancreas and quantify BCM [2]. A validated PET imaging marker for BCM could greatly improve our understanding of diabetes etiology and enable new endpoints in therapy development.

A transmembrane G-protein-coupled receptor (GPR44, also known as CRTH2, PTGDR2, or CD294) that binds to endogenous prostaglandin D2 was recently identified as a biomarker for monitoring BCM by proteomic and transcriptomic screening [3]. Specifically, GPR44 is highly expressed in beta cells but not in other exocrine and endocrine cells in the human pancreas, nonhuman primates, and pigs [4]. Activation of GPR44 by endogenous prostaglandin D2 was posited to inhibit insulin secretion—a hypothesis supported by increased insulin secretion in response to administration of GPR44 inhibitors in vitro and in vivo. However, oral administration of a GRP44 inhibitor showed no major impact on insulin secretion in patients with type 2 diabetes [5] but demonstrated improvement of islet function under inflammatory and hyperglycemic stress [6]. GPR44 is also expressed on certain immune cells, e.g., eosinophils and T-helper type 2 cells (Th2), which are involved in asthma and are related to eosinophil activation. Thus, inhibition of GPR44 has been well studied in inflammatory processes responsible for allergy and asthma [7]. Numerous GPR44 antagonists have therefore been developed to treat asthma and other allergic diseases, and a few of these have entered late clinical phase drug development [8].

Previous evaluation of GPR44 antagonists [^3^H]AZD3825 and [^11^C]AZ12204657 showed promising results, indicating that GPR44 could be a suitable target for imaging BCM [9,10]. MK-7246 is a GPR44 antagonist originally developed to treat respiratory diseases [11]. It was reported to selectively bind to GPR44 in a reversible manner and possesses high affinity and good pharmacokinetic properties [12]. Our group previously radiolabeled MK-7246 with carbon-11. Initial preliminary preclinical evaluation showed promising results in binding, biodistribution, and dosimetry tests [13]. Herein, we describe an improved method to synthesize [^11^C]MK-7246 and further evaluate it as a marker for GPR44 in a pig model and in cell lines.

## 2. Materials and Methods

### 2.1. General Information

All reagents were purchased from Sigma-Aldrich (analytical grade or higher) and used without further purification, unless stated otherwise. Helium and nitrogen (with 0.05% oxygen) gas were purchased from AGA. Crimp neck conical reaction vials (0.9 mL) were purchased from VWR and the seal (1.5 mm thickness, 11 mm diameter, aluminum crimp cap, silicone/PTFE septum) was purchased from Scantec Nordic.

### 2.2. In Silico Docking 

**Figure 1:**
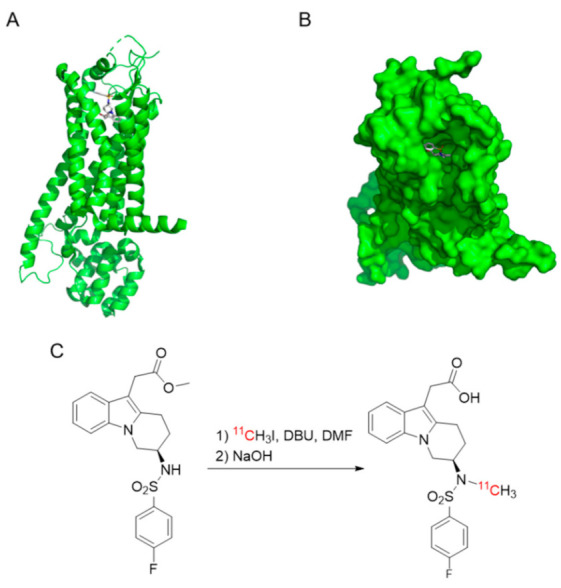
(**A**,**B**) Docking structure of MK-7246 with GPR44 (−10 kcal/mol binding affinity). (**C**) Synthesis of [^11^C]MK-7246.

The in silico structure of human GPR44 was obtained from the RCSB Protein Data Bank (6D27.pdb) (Figure 1A,B). The structure of MK-7246 was exported as an mdl file from ChemDraw 18.2 (PerkinElmer) and converted to pdb format by the OpenBabel online tool.

Docking of MK-7246 to GPR44 was performed in the visualization software PythonPrescription PyRx 0.8 using molecular docking program AutoDock Vina. The in silico assessment of binding affinity (ΔG, in kcal/mol) was obtained from the optimal docking score. The docked molecules were visualized in the software PyMOL (Schrödinger).

### 2.3. Radiochemistry

The radiosynthesis was conducted in Tracer Production System (TPS), which was built inhouse for automation. Preparative high-performance liquid chromatography (prep-HPLC) was performed using an Agilent 1260 Infinity II pump, Agilent 1260 Infinity II UV detector, Bioscan Flow-count PMT radiodetector, TPS autosampler, and an ACE 5 SuperC18 column (150 × 10 mm). Analytical HPLC was performed using an Agilent 1290 Infinity II pump, Agilent 1290 Infinity II UV detector, Flow-count PMT radiodetector, and a Chromolith Performance RP-18e column (100 × 4.6 mm). The mobile phase of prep-HPLC was 53% acetonitrile in ammonium formate (50 mM, pH 3.5) at a flow rate of 5 mL/min. The mobile phase of analytical HPLC was acetonitrile and water (with 0.1% trifluoroacetic acid) at a flow rate of 4 mL/min, varying the concentration of acetonitrile from 5 to 73% over 8 min. All radiochemical yields (RCYs) were calculated based on [^11^C]methyl iodide and the activity in the formulated product (decay corrected). The radiochemical purity (RP) and the molar activity were assessed by analytical HPLC with a sample withdrawn from the formulated product solution at the end of synthesis (EOS). The identity of the labeled products was assessed by spiking the sample with nonradioactive MK-7246.

Cyclotron-produced [^11^C]carbon dioxide (approximately 20 µAh of irradiation) was transferred to a solution of 300 μL LiAlH_4_ (0.1 M) in tetrahydrofuran (THF) in a glass vessel. The THF was evaporated at 80 °C for 2 min with a nitrogen flow of 160 mL/min. Then, 600 μL of hydriodic acid (57%) was added and the mixture was heated at 130 °C for 1 min with both the inlet and outlet of the vessel closed. The formed [^11^C]methyl iodide was transferred by nitrogen gas at a flow of 10 mL/min over a column packed with phosphorous pentoxide and Ascarite into a reaction vial which contained 1 mg *N*-desmethyl-*O*-methyl MK-7246 precursor and 1 μL 1,8-diazabicyclo[5.4.0]undec-7-ene (DBU) in 200 μL dimethylformamide (DMF) (Figure 1C). The reaction vial was heated at 110 °C for 4 min, with one shake per min, followed by the addition of 200 μL NaOH (1 M) and deprotection at 75 °C for 4 min. After cooling, the reaction mixture was neutralized with 400 μL HCl (1.0 M) and injected into the prep-HPLC system.

The fraction at 9.1 min was collected in a vial containing 20 mg ascorbic acid (to prevent radiolysis) in 0.5 mL water. The mixture was diluted with water (20 mL) and passed over a solid phase extraction (SPE) cartridge (C18 Plus Light, Waters). The trapped product was washed with water (20 mL) and eluted with ethanol (0.4 mL) followed by PBS (4 mL). Lastly, the formulated product was transferred to the capped product vial through a sterile filter (Millex-GV, 0.22 µm, 33 mm, Millipore).

### 2.4. Cell Lines

The GPR44-overexpressing Chinese hamster ovarian cell line (CHO-K1) was purchased from PerkinElmer and cultured according to the provider’s guidelines in Ham’s F-12 (Biowest, Riverside, MO, USA), 10% FBS (Sigma), 0.4 mg/mL G418 (VWR Life Science), and 2 nM L-Glutamine (Merck Millipore, Darmstadt, Germany). Nontransfected CHO-K1 cells, used as negative control, were purchased from ATCC and cultured in Ham’s F-12 (Biowest, Riverside, MO, USA), 10% FBS (Sigma), and 1% Penicillin/Streptomycin (Merck Millipore, Darmstadt, Germany).

The CHO-K1 cells were first detached using trypsin 0.25% EDTA before they were washed with PBS pH 7.4 and centrifuged into a pellet, prior to embedding into optimal cutting temperature (OCT) compound (Q Path mounting media, VWR) and freezing at −80 °C. Slices of 20 µm thickness were prepared on cryotome (Cryostat NX70, Thermo Fisher) before mounting on SuperFrost Plus (Thermo Fisher) slides and stored at −30 °C prior to the experiment.

### 2.5. In Vitro Autoradiography of Cell Pellets

Frozen sections of CHO-K1 cells overexpressing GPR44 and nontransfected CHO-K1 cells were first immersed in PBS for 10 min at room temperature (RT) before 40 min of incubation with 0.2 MBq/mL of [^11^C]MK-7246 (molar activity between 400 and 600 GBq/µmol) in PBS at RT for a final concentration ranging from 0.3 to 0.5 nM. Nonradioactive MK-7246 at a concentration of 2 µM (dissolved in PBS with 10% glucose and 10% DMSO) was used in parallel to ascertain nonspecific binding.

A series of two cold washes in PBS and one in deionized water (all for 1 min) were performed after the incubation. Samples were dried and left for exposure on the phosphorimager plate (Fujifilm, Tokyo, Japan) for 3 half-lives (60 min) of the radionuclide.

Drops (20 µL) of reference solution on absorbent paper, cross-measured in a well counter (Uppsala Imanet AB, Uppsala, Sweden), were also included to enable quantification of the autoradiograms. The resulting digital image was obtained from an Amersham Typhoon phosphorimager (GE Healthcare, Chicago, IL, USA). A total of 3 assays were performed, each with a different batch of radioligand. In each assay, a minimum of two duplicate sections were used.

Autoradiograms were analyzed using ImageJ (National Institute of Health). The cell sections and the references were delineated briefly. The uptake values were corrected for background counts and converted to Bq/mm^2^ (through the references). The binding was further corrected to Bq/mm^3^ using the thickness of the section (20 µm), and finally to fmol/mm^3^ using the molar activity ascertained at the start of the experiments.

### 2.6. Animal Handling

A total of *n* = 6 (3 males and 3 female) pigs (Yorkshire x Swedish Landrace x Hampshire) with weight of 25–30 kg were provided by Hedenstierna lab and transported by car from the farm on the day of the experiment. All of the performed procedures were in accordance to the ARRIVE guidelines for animal research and approved by the Animal Research Ethical Committee of the Uppsala Region, with consideration for the Uppsala University guidelines for animal research (C32/15).

### 2.7. In Vivo Biodistribution with Dynamic PET/CT in Pig

For each of the pigs, a baseline scan was performed (*n* = 6). A second scan was then performed following administration of a high dose of GPR44 inhibitor (*n* = 5).

Anesthesia was given intravenously through the ear with a combination of ketamine, fentanyl, and midazolam before placing the pigs under assisted ventilation through an endotracheal tube. An arterial catheter was placed in the carotid artery and a venous catheter was placed in the jugular vein.

The pigs were connected to an intratracheal ventilator and a central venous catheter for tracer, blocking drug, and contrast infusion. The pigs were also connected to an arterial catheter through the side branch of the carotid for blood sampling purposes. Glucose levels were monitored regularly using blood samples and a glucose meter. A computed tomography (CT) acquisition for attenuation and localization was first performed (100 kV, 80–400 mA, noise index 10, rotation 0.5′, full spiral, slice thickness 3.75 mm, pitch 0.98:1, recon diameter 50 mm) before injecting approximately 10 MBq/kg of [^11^C]MK-7246 dissolved in 2 mL of PBS. A dynamic PET imaging of 90 min was then acquired using the following parameters: 33 frames of 12 × 10′’, 6 × 30′’, 5 × 2′, 5 × 5′, 5 × 10′, VPFX-S, 3 i/16 s, 256 × 256 pixels, 3 mm post filter, 50 cm diameter zoom. At the end of the dynamic PET scan, a whole-body CT (100 kV, 80–400 mA, noise index 10, rotation 0.5′, full spiral, slice thickness 3.75 mm, pitch 0.98:1, recon diameter 50 mm) and a static PET (VPFX-S, 3 i/16 s, 256 × 256 pixels, 3 mm post filter, 50 cm diameter zoom) were performed. There was a waiting period of at least 2 h (approximately 6 half-lives of carbon-11) before engaging the following PET acquisition with nonradioactive MK-7246 compound so that the signal from the previous injection would be negligible. Nonradioactive MK-7246 (1 mg/kg) dissolved in 100 mL of PBS containing 10% glucose was injected intravenously by bolus using a filtered syringe approximately 30 min prior to starting the second PET/CT scan using the same protocol described previously.

Once the PET/CT dynamic scan was finished, a pancreas contrast CT acquisition using 3 mL/kg Omnipaque (GE Healthcare), bolus tracking with 100 HU threshold and region of interest placed in aorta, 15 s monitor delay for arterial phase, and 60 s delay for venous phase with 3.5 mL/s flow was performed. All of the sequences described previously were performed in a Discovery MI PET/CT scanner (GE Healthcare, Chicago, IL, USA).

PET data were analyzed with manual segmentation of the pancreas, spleen, and bone marrow (liver and kidney were not reported in the results) on sequential transaxial projections using the PBAS modeling tool (PMOD technologies LLC, Zurich, Switzerland). The arterial concentration was determined by selecting the hottest pixel in the lumen of the descending aorta, on approximately 10 sequential transaxial sections.

The uptake in Bq/cc was converted to standardized uptake values (SUV) by correcting for the amount and time of administered radioactivity (in MBq) and the weight (in kg) of each pig. The uptake in tissue was presented as the tissue to aorta ratio from 60–90 min.

The radioactive dose to organs was obtained via extrapolation of the pig data to human, using reference measurements from the International Commission on Radiological Protection publication 89 (ICRP89) annals and from the Medical Internal Radiation Dose (MIRD) model values described by Cristy and Eckerman (1987) [14,15]. The residence time (RT), representing the number of radioactive particles disintegrated per injected dose, was obtained from the area under the curve (AUC) of the time–activity curve. The AUC was calculated using the trapezoid method for 8 time points and the integral of the tail area, assuming a monoexponential decay of the compound starting from the last measurable time. Dose to each individual organ, effective dose (ED), and effective dose equivalent (EDE) were obtained from the software OLINDA/EXM 1.1 (Organ Level Internal Dose Assessment Code, Vanderbilt University, Nashville, TN) using the averaged RT according to a reference phantom model based on the ICRP89.

### 2.8. Statistics

All statistical analysis was performed on GraphPad Prism (version 8.3.1). Differences between groups were assessed by unpaired Student’s *t*-test for the in vitro studies and paired Student’s *t*-test for the in vivo studies, using *p* < 0.05 as the limit of significance.

## 3. Results

### 3.1. Radiochemistry

The radioactivity of the formulated [^11^C]MK-7246 was 800 ± 152 MBq (entries 1–7) at EOS when using 1 mg of precursor (for pig delivery) and was decreased to 522 ± 89 MBq (entries 8–13) at EOS when using 0.5 mg of precursor (for autoradiography delivery) (Table 1). Accordingly, the RCY was 16 ± 2% (entries 1–7) when using 1 mg of precursor and was decreased to 9 ± 2% (entries 8–13) when using 0.5 mg of precursor. The RP was initially 85 ± 4% (entries 1–6) and was increased to 92% (entry 7) with the addition of ascorbic acid in the formulation process. The RP was further improved to 95 ± 1% (entries 8–13) when using 0.5 mg of precursor. The molar activity was 564 ± 152 GBq/µmol (entries 1–7) at EOS when using 1 mg of precursor and was slightly decreased to 482 ± 196 GBq/µmol (entry 8–13) at EOS when using 0.5 mg of precursor.

### 3.2. In Vitro Autoradiography

**Figure 2:**
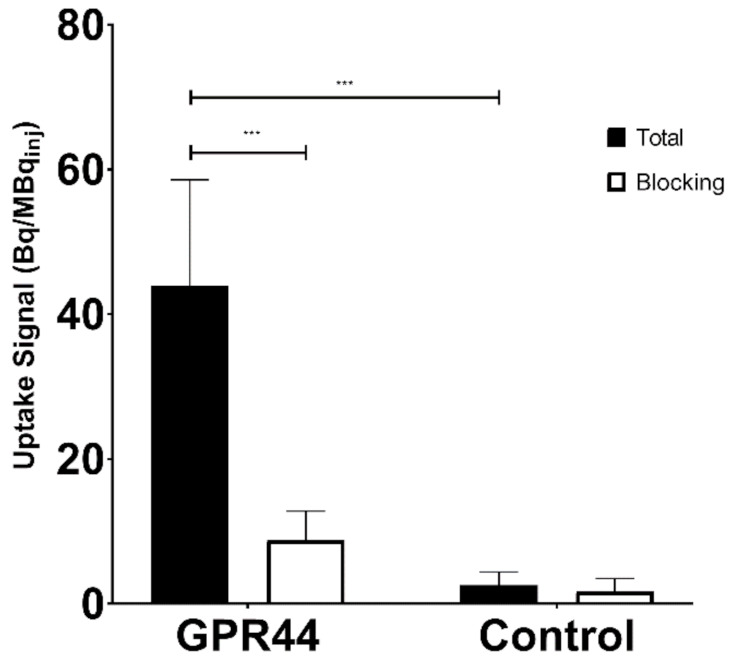
Receptor signal to injected dose ratio (Bq/MBq_inj_) and blocking effect in the GPR44-overexpressing CHO-K1 cell line, compared with the nontransfected CHO-K1 cell line (*** *p* < 0.0001).

GPR44-overexpressing CHO-K1 frozen sections showed a significantly higher binding of [^11^C]MK-7246 (uptake signal = 43.91 ± 14.67 Bq/MBq_inj_, *n* = 12) compared with the nontransfected CHO-K1 negative control (uptake signal = 8.74 ± 1.83 Bq/MBq_inj_, *n* = 8) (*p* < 0.05). As expected, the binding could be blocked in the CHO-K1 sections overexpressing GPR44 by coincubation of the nonradioactive MK-7246 compound (*p* < 0.05) (Figure 2).

### 3.3. In Vivo Biodistribution with Dynamic PET/CT in Pigs

**Figure 3:**
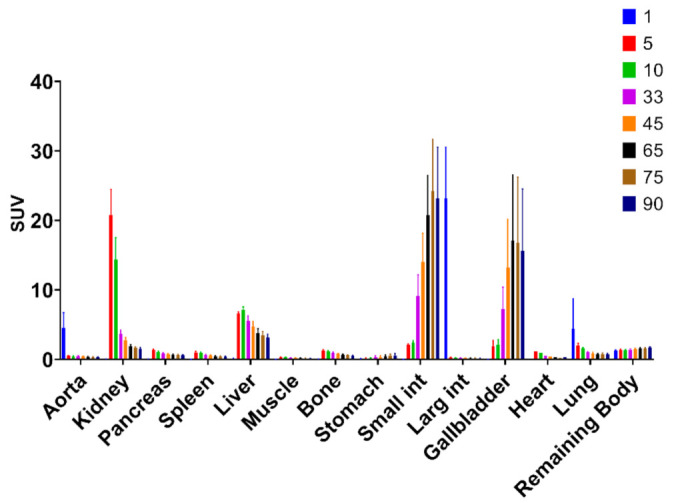
Biodistribution of [^11^C]MK-7246 in each individual organ over time (min) with 60–90 min as the optimal biodistribution window.

The biodistribution of [^11^C]MK-7246 demonstrated rapid uptake and washout from most tissues, except kidney, liver, and small intestines, with continuous uptake signal attributed to excretion (Figure 3).

**Figure 4:**
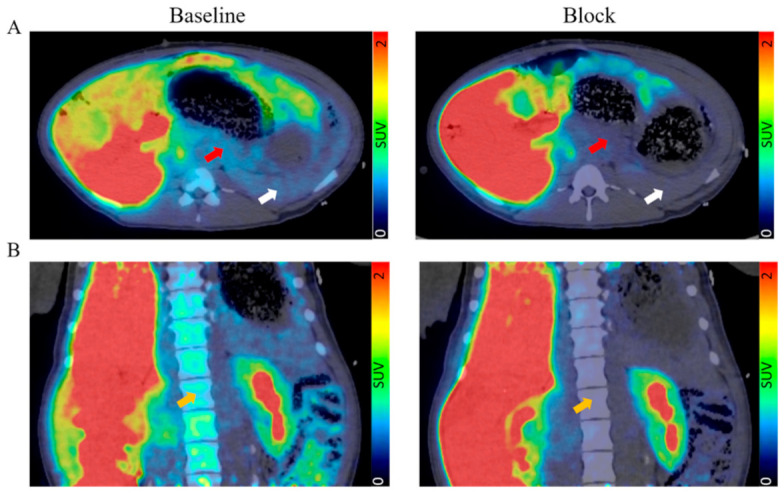
(**A**) Transversal and (**B**) coronal view of fused PET/CT scan with the averaged signal from 60–90 min. Red arrow indicates pancreas, white arrow indicates spleen, and orange arrow indicates bone marrow.

No adverse reactions were seen in any of the pigs to either [^11^C]MK-7246 nor the nonradioactive compound. The pancreas was clearly identified and did not exhibit spillover from adjacent tissues (Figure 4A). The spleen could also be clearly distinguished due to the expression of GPPR44 in pigs. The bone marrow was visible as well, likely due to resting immune cells expressing GPR44 (Figure 4B).

**Figure 5:**
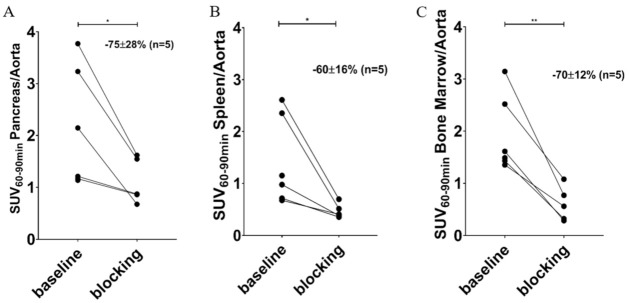
Averaged SUV from 60–90 min normalized to the aorta of (**A**) pancreas, (**B**) spleen, and (**C**) bone marrow (* *p* < 0.05; ** *p* < 0.01).

The binding of [^11^C]MK-7246 was calculated by averaging the signal from 60 to 90 min and normalizing this to the aorta signal. The pancreas uptake was well above the background and blood signal (SUV pancreas/aorta ratio = 2.30 ± 1.18, *n* = 5). Importantly, the binding could be blocked by preadministration of a GPR44 antagonist (SUV pancreas/aorta ratio following block = 1.11 ± 0.44, *n* = 5) and the decrease in each individual pig due to blocking was, on average, 75% (*p* < 0.05) (Figure 5A). Similarly, we observed blockable binding in pig spleen (Figure 5B) and bone marrow (Figure 5C).

**Figure 6:**
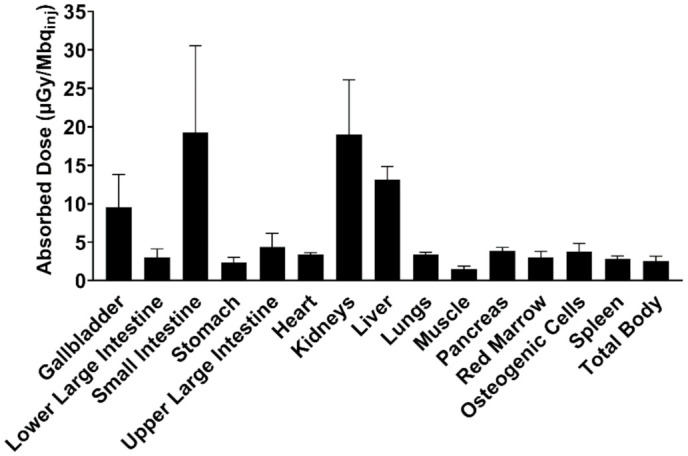
Human predicted dosimetry profile of [^11^C]MK-7246 extrapolated from pig biodistribution data, expressed as the absorbed dose per unit of administered radioactivity.

The absorbed dose for each individual organ was highest for the small intestine, kidney, and liver (Figure 6), with an average whole-body ED of 3.60 ± 0.72 µSv/MBq_inj_, limiting the maximum annual dose of [^11^C]MK-7246 to 2777 MBq (i.e., approximately 8 PET scans annually).

## 4. Discussion

**Figure 7:**
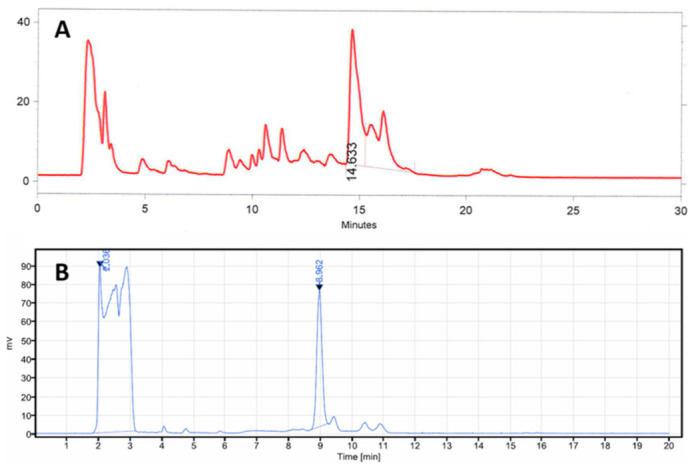
Prep-HPLC radio chromatograph of [^11^C]MK-7246 using (**A**) the previous method and (**B**) the new method described in this paper.

The radioactivity of [^11^C]MK-7246 was significantly improved from 270 ± 120 MBq to 800 ± 152 MBq compared with our previous publication using a similar starting activity. The increased RCY was partially due to a change from vortex evaporation to SPE for the removal of the HPLC eluent and the product formulation. We also concluded that the previously used sodium hydride base in the methylation reaction negatively affected the deprotection step, introducing several side products as observed in the prep-HPLC chromatogram (Figure 7A). Focusing on soluble bases, we first tried a change to the milder base triethylamine but it did not give any product. DBU however facilitated the ^11^C-methylation and allowed the subsequent deprotection step to proceed with improved selectivity. The optimal quantity of DBU was 0.1 µL (0.27 equivalents). The switch to a new preparative column (ACE 5 SuperC18) also improved the chromatography compared with the previous method (Figure 7B).

The deprotection of the [^11^C]MK-7246 methyl ester was then optimized by reducing the concentration of added sodium hydroxide by half, which resulted in a higher RCY. When the optimized reaction conditions were applied with 1.0 mg of precursor (a reduction from the previously used 1.5 mg) ~800 MBq of product was obtained. Even with 0.5 mg of precursor, enough product (~400 MBq) was obtained to conduct autoradiography experiments. With the increased radioactivity, we observed that the purity of [^11^C]MK-7246 in the formulated product decreased, seemingly due to radiolysis during the formulation process. This issue was mended by adding 20 mg of ascorbic acid predissolved in 0.5 mL of water to the collected HPLC fraction. The molar activity of [^11^C]MK-7246 was also improved from ~100 GBq/µmol to ~500 GBq/µmol. This increase could be explained by more thorough flushing of the TPS nitrogen process gas prior to the synthesis start, purging atmospheric CO_2_ from the idle tubing. The high amount of molar activity was probably due to the HPLC UV lamp detection threshold (especially for entries 6 and 10 in Table 1—the UV signal of MK-7246 was so low that it could not be detected by HPLC.)

In vitro frozen section autoradiography demonstrated a strong binding to the GPR44-overexpressing CHO-K1 cell line but not in the control nontransfected CHO-K1 cell line (no target receptor present for binding). More importantly, specificity of the binding was repeatedly demonstrated through a decrease in binding after coincubation with excess nonradioactive MK-7246, after which the radiolabeled [^11^C]MK-7246 displayed the same binding properties as the unlabeled nonradioactive MK-7246. 

In the in vivo dynamic PET/CT pig imaging, the biodistribution demonstrated mainly hepato-biliary excretion with a clearly defined pancreas in agreement with previous studies. The optimal biodistribution window for [^11^C]MK-7246 was set between 60–90 min when the hepato-biliary elimination of the radiopharmaceutical had occurred, as shown by the sharp decrease in liver signal corroborated with the simultaneous increase in the gallbladder and small intestine signals.

The pancreatic binding was low (SUV ≈ 1) but clearly visible above the background, consistent with the notion of dispersed beta cells in the pancreatic volume but each with strong expression of the GPR44 receptor. The spleen signal could potentially pose a spill-in issue due to the close anatomical proximity to the pancreas, but previous results using the similar GPR44-targeting radioligand [^11^C]AZD12204657 showed low binding in human spleen sections, suggesting that the relatively strong splenic signal might be a species-dependent phenomenon.

Importantly, the in vivo uptake in the pancreas, spleen, and bone marrow could also be saturated by nonradioactive MK-7246, indicating receptor-mediated binding. Leftover binding signal after blocking in both the frozen section autoradiography and pig PET/CT imaging can be explained by the poor solubility of the non-radioactive MK-7246 and further assessment of specificity may focus on the use of GPR44 inhibitors from other molecular classes (e.g., Fevipiprant, AZD1981). Well-defined subsets of immune cells have been shown to express GPR44, eosinophils, and Th2 [16]. Their involvement in asthma provided the impetus for interest in GPR44 antagonists such as MK-7246. Neither eosinophils nor Th2 cells are known for contributing to inflammation of the pancreas in type 1 or type 2 diabetes and should not provide a confounding signal in the context of pancreatic beta-cell imaging. However, low background signal could be observed in the lungs and we expect radiolabeled MK-7246 to be suitable for in vivo assessment of eosinophils in lung inflammation, including COVID-19.

MK-7246 has been shown to exhibit a suitable safety profile in phase I clinical studies for oral dosing up to 320 mg [17] and, accordingly, no pig demonstrated any adverse symptoms following baseline administration of [^11^C]MK-7246 in this study. Based on the radiochemistry data demonstrated here, we predict the amount of [^11^C]MK-7246 administered intravenously to human in clinical studies by PET will be in the low microgram range, which is far below any dose causing pharmacological side effects. Furthermore, this study demonstrated positive extrapolated human dosimetry data for [^11^C]MK-7246, revealing a low absorbed radioactive dose—especially in sensitive tissues such as the bone marrow—implying that [^11^C]MK-7246 at microdose levels is likely a safe compound for repeated PET imaging in humans. The obtained results are in accordance with previous human dosimetry data extrapolated from rats. Pigs showed similar liver and intestinal excretion patterns to rats but exhibited greater retention in pancreas than rats did, which was expected due to the presence of GPR44 in pig beta cells.

Ex vivo autoradiography assessment of the pig pancreas to demonstrate targeting of the islets in particular was not feasible due to the short half-life (20 min) of the carbon-11 radionuclide. Radiolabelling of MK-7246 with a longer-lived radionuclide would potentially enable ex vivo studies of tissue binding. Since MK-7246 has a fluorine atom at the para position of the phenyl ring (Figure 1C), it can theoretically be radiolabeled with fluorine-18, which has a longer half-life of 110 min and a lower positron energy, which will lead to improved spatial resolution. This is an important feature of the MK-7246 compound compared with previously radiolabeled GPR44 inhibitors such as AZ12204657, which lacks a fluorine atom for isotopic labelling. An induced diabetes pig model with, e.g., streptozocin, would have been desirable but was not achievable due to the low streptozocin sensitivity in pigs compared with rodents [18]. Further studies are ongoing to implement a GMP-compliant production method for translation into clinical evaluation.

High-throughput approaches such as proteomics and transcriptomic screenings have identified diverse surface biomarkers for pancreatic islets other than GPR44, for instance dipeptidyl peptidase 6 (DPP6) or FXYD2γa. Based on these findings, radiolabeled probes such as technetium-99 or gallium-68 labeled nanobody have been developed targeting DPP6 for single-photon emission computed tomography (SPECT) and PET, respectively [19]. However, SPECT offers poorer resolution compared with PET and, despite well-described chemistry and widely accessible gallium-68 generators, short positron range radioisotopes such as carbon-11 or fluorine-18 offer better spatial resolution.

Gd-DOTA-conjugated phage display-derived peptide (P88) targeting FXYD2γa for magnetic resonance contrast imaging (MRI contrast) provides excellent soft tissue contrast and a nonradioactive alternative imaging method [20]. However, the low sensitivity of this technique often requires a relatively large amount of contrasting agent—associated with potential, although rare, adverse effects. Lack of precise quantification with MR contrast is also a downside in BCM imaging.

## 5. Conclusions

The synthesis method of [^11^C]MK-7246 was improved with increased radioactivity and RCY. The preclinical in vitro and in vivo assessment of [^11^C]MK-7246 underlined a robust binding property to GPR44 with promising biodistribution, kinetics and a likely safe dosimetry for future GPR44-directed compound development. Additional studies involving longer-lived radionuclides on pancreatic islets are, however, necessary to further support its potential application in BCM imaging.

## Figures and Tables

**Table 1:** Summary of radiochemistry results. Ascorbic acid was added in entries 7–12. A_product_ = radioactivity of product, RCY = radiochemical yield (decay corrected), RP = radiochemical purity (decay corrected), A_m_ = molar activity (at end of synthesis).

Entry	Precursor (mg)	A_product_ (MBq)	RCY	RP	A_m_ (GBq/µmol)
1	1.0	919	16%	81%	684
2	1.0	507	17%	86%	542
3	1.0	1031	18%	80%	513
4	1.0	750	17%	85%	588
5	1.0	735	14%	88%	769
6	1.0	839	14%	87%	n/a
7	1.0	818	16%	92%	284
8	0.5	436	6%	96%	324
9	0.5	400	9%	97%	272
10	0.5	631	9%	94%	n/a
11	0.5	554	11%	94%	759
12	0.5	591	9%	94%	572

## Data Availability

The datasets generated and analysed during the current study are available from the corresponding author on reasonable request.
